# Effects of Explosive vs. Strength Resistance Training on Plantar Flexor Neuromuscular and Functional Capacities in Institutionalized Older Adults: A Randomized Controlled Trial

**DOI:** 10.3390/jfmk9040261

**Published:** 2024-12-07

**Authors:** Elmoetez Magtouf, Nicolas Peyrot, Yosra Cherni, Oussema Gaied Chortane, Jonathan Jolibois, Abderrahmane Rahmani, Wael Maktouf

**Affiliations:** 1 Research Laboratory (LR23JS01) «Sport Performance, Health & Society», Higher Institute of Sport and Physical Education of Ksar Saîd, University of “La Manouba”, Tunis 2010, Tunisia; elmoetez_bellah.magtouf.etu@univ-lemans.fr (E.M.); oussama.gaeid@gmail.com (O.G.C.); 2 Laboratory “Movement, Interactions, Performance” (UR 4334), Department of Sport Sciences, Faculty of Sciences and Technologies, Le Mans University, 72000 Le Mans, France; nicolas.peyrot@univ-lemans.fr (N.P.); abdel.rahmani@univ-lemans.fr (A.R.); 3 Intercommunal Health Center of Sarthe et Loir, 72000 Le Mans, France; jjolibois@pole-pssl.fr; 4 School of Kinesiology and Exercise Sciences, Faculty of Medicine, University of Montréal & UHC Sainte Justine Research Center, Montreal, QC H3T 1J4, Canada; yosra.cherni@umontreal.ca; 5 Bioengineering, Tissues and Neuroplasticity, UR 7377, Faculty of Health/EPISEN, University of Paris-Est Créteil, 94010 Créteil, France

**Keywords:** rate of force development, maximum strength, gait speed, elderly, strength training

## Abstract

Objectives: To compare the effects of explosive and strength resistance training on neuromuscular and functional parameters in older adults and to analyze the relationship between changes in walking speed and improvements in plantar flexor (PF) neuromuscular parameters following interventions. Methods: In total, 40 participants were randomly assigned to either an explosive resistance training group (EXG, n = 18; age = 80.41 ± 10.12 years; body mass index = 22.89 ± 2.77 kg/m^2^) or a strength resistance training group (STG, n = 22; age = 82.89 ± 5.32 years; body mass index = 23.81 ± 3.45 kg/m^2^). Both groups participated in the same PF resistance training regimen (three sessions per week for 12 weeks), engaging in identical exercises. However, EXG performed three to five sets of 12 to 14 repetitions at 40% to 45% of one-maximal repetition with a rapid concentric phase and a 3 s eccentric phase, while the STG performed three to four sets of 6 to 7 repetitions at 80% to 85% of one-maximal repetition, with both phases lasting approximately 3 s. Before and after the interventions, gait speed (m/s) was assessed using a 10 m walking test, and relative maximal force (Relative Fmax, N/kg) was evaluated during maximal voluntary isometric contraction of PF. From the force–time curve, early (0–50 ms) and late (100–200 ms) rates of force development (RFD) were extracted from the linear slopes (Δ force/Δ time). Results: Gait speed significantly improved in both groups (*p* < 0.05). However, the improvement was more pronounced in the EXG compared to the STG (*p* < 0.05). Relative Fmax showed a more significant increase in the STG than in the EXG (*p* < 0.05). Moreover, a significant 10% increase in early RFD in the STG and a 20.1% increase in the EXG were observed (*p* < 0.05). The improvement in early RFD was greater in the EXG (*p* < 0.05). Additionally, late RFD improved significantly only in the EXG (+20.4%, *p* < 0.05). Conclusion: Explosive resistance training appears particularly effective in improving the ability to rapidly generate force, which is essential for many daily activities requiring explosive movements and quick responses.

## 1. Introduction

Resistance training has long been recognized as one of the most effective interventions to counteract sarcopenia and improve neuromuscular function in older adults [1,2]. Traditionally, strength-based resistance training—characterized by slow and controlled repetitions at moderate to high loads—has been widely advocated. This form of training enhances maximal strength and muscle mass, which are essential for maintaining functional independence [3]. However, recent evidence suggests that explosive resistance training, which emphasizes rapid force production during the concentric phase of movement, may confer additional benefits [4]. Explosive resistance training has been shown to improve not only maximal strength but also muscle power, an important predictor of functional capacity and fall risk in older adults [5]. Power-oriented adaptations are particularly relevant as they reflect the ability to generate force quickly, which is crucial for tasks like recovering balance and preventing falls [6]. Despite this, the relative effectiveness of explosive resistance training compared to traditional strength resistance training in improving key neuromuscular outcomes in older adults remains an area of ongoing research.

Several studies have highlighted a significant correlation between decreased ankle plantar flexor (PF) force production capacities and functional abilities [2,3]. For instance, Cattagni et al. [4] observed that within a group of 30 older adults, the maximal torque of the PF was negatively correlated with the displacement of the center of pressure during static postural balance (r = −0.77). Furthermore, they demonstrated that 90% of individuals with maximal PF torque less than 3.1 Nm/kg had previously experienced falls, whereas 85% of those exceeding this value had not. Therefore, maximal PF force production capacity could be considered a predictive parameter for fall risk [5]. However, in older adults, the ability to avoid a fall depends on both maximal muscle strength and the rate of force development (RFD), which reflects the speed at which submaximal force can be generated [7]. RFD is often divided into early (0–50 ms) and late (100–200 ms) phases, each influenced by distinct neuromuscular mechanisms [8,9]. The early phase depends on motor unit recruitment, firing rates, and intrinsic muscle properties, such as calcium dynamics, while the late phase involves force transmission through the connective tissues [10]. Improving RFD is therefore a key objective in training programs targeting walking ability, balance recovery, and fall prevention [11,12].

Recent studies have demonstrated that explosive resistance training has a greater impact on RFD compared to strength resistance training [13]. For example, Cadore et al. [14] showed significant improvements in muscle power, strength, and dual-task performance, following explosive resistance training in frail, institutionalized older adults [15]. Additionally, explosive resistance training may enhance not only muscle force production but also functional outcomes such as walking speed, a critical marker of mobility, and overall health in older adults [16]. However, there remains a paucity of research comparing the specific effects of explosive and strength resistance training on both early and late RFD of the ankle plantar flexors (PF), a muscle group essential for balance, gait, and fall prevention [17].

Given the central role of the ankle PF in functional tasks and the established relationship between PF strength, RFD, and fall risk [14,18], it is imperative to evaluate the relative benefits of explosive versus strength resistance training in this context. Additionally, gait speed is a critical outcome, as it serves as a strong predictor of morbidity and mortality in older adults [19]. While strength resistance training is effective for improving PF maximal force, explosive resistance training may provide superior benefits by targeting both early and late RFD, thereby enhancing dynamic functional capacities.

The primary objective of this study was to compare the effects of explosive versus strength resistance training on maximal strength, early and late RFD of the PF, and gait speed in institutionalized older adults. We hypothesize that explosive resistance training results in greater improvements in PF neuromuscular and functional parameters, compared to strength RT. The second objective was to analyze the relationship between changes in walking speed and improvements in PF neuromuscular parameters following interventions. We hypothesize that improvements in gait speed are correlated with increased neuromuscular parameters of PF.

## 2. Materials and Methods

### 2.1. Study Design

This study strictly adhered to the Consolidated Standards of Reporting Trials guidelines and was designed as a prospective, single-blinded, controlled, randomized trial. Participants were randomly assigned to either an explosive resistance training group (EXG) or a strength resistance training group (STG), using a computer-generated randomization sequence stratified by age, sex, and baseline PF strength to ensure balanced group allocation and minimize potential confounding variables. The study was conducted in accordance with the ethical principles outlined in the Declaration of Helsinki and received approval from the local Ethics Committee of the Intercommunal Health Center of Sarthe et Loir (protocol code 012, approved on 5 January 2024). The committee also approved the study protocol, patient information sheet, and informed consent form.

### 2.2. Recruitment

**Figure 1:**
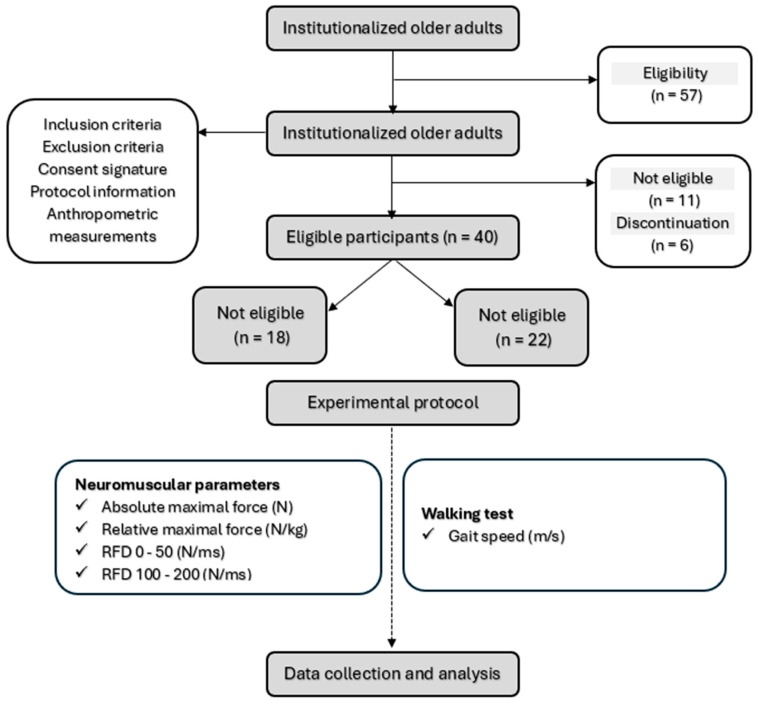
Study design.

The recruitment process consisted of a three-week recruitment period, followed by a one-week screening phase (Figure 1). Participants were recruited from a retirement home located in Sablé-sur-Sarthe (France) between 15 January and 15 February 2024. The eligibility of each participant was meticulously verified by the medical staff before randomization. 

**Inclusion Criteria**

Participants were eligible for the study if they fulfilled the following criteria:

- They were aged 65 years or older;
- They could walk independently without technical assistance (e.g., canes, walkers) or help from another person;
- They were able to communicate verbally effectively with the research team.

**Exclusion Criteria**

Participants were excluded if they had the following conditions:

- They had neurological or cognitive disorders.
- They suffered severe cardiovascular diseases.
- They experienced significant musculoskeletal issues in the lower limbs.
- They were taking medications that could affect the neuromuscular or functional tests.

Eligible participants were randomized into one of two groups: EXG or STG. The randomization list was generated using a computer algorithm by an independent statistician. At the end of the screening process, the investigator, who was blinded to the treatment assignment, obtained a unique randomization number for each participant.

### 2.3. Evaluation Protocol

Both groups underwent the same assessments before and after the intervention. All assessments were conducted in a clinical examination room under consistent environmental conditions, supervised by a blinded evaluator who was unaware of the group affiliation of the participants. Prior to the assessments, participants received a standardized set of verbal instructions to ensure familiarity with the procedures. All evaluation sessions were preceded by a standardized warm-up (5 min of cycling and submaximal PF contractions) and cool-down (static stretching).

#### 2.3.1. Anthropometric Parameters

The body mass and height of the participants were precisely measured using a digital floor scale and a wall-mounted stadiometer, respectively. Body mass index (kg/m^2^) was subsequently calculated. Lean body mass was measured using bioelectrical impedance (Tanita; SC 240, Amsterdam, The Netherlands) [20]. During the measurement, participants were instructed to stand still, with their arms slightly away from their body and their legs not touching, while their feet were positioned on the electrodes of the bioelectrical impedance. All measurements were conducted in the morning, with participants fasting and having avoided any physical activity for 24 h prior.

#### 2.3.2. Gait Speed

Gait speed (m/s) was assessed over a 20 m flat surface, with measurements taken between the 5th and 15th meters to exclude acceleration and deceleration phases [21]. Participants were instructed to walk at their usual pace and attempt to reach their maximum speed between the 5th and 15th meters. A stopwatch was used to measure the time required to walk the 10 m distance. The average walking speed (m/s) was then calculated using the following formula:

Walking speed (m/s) = distance/time,(1)

#### 2.3.3. Neuromuscular Parameters

**Figure 2:**
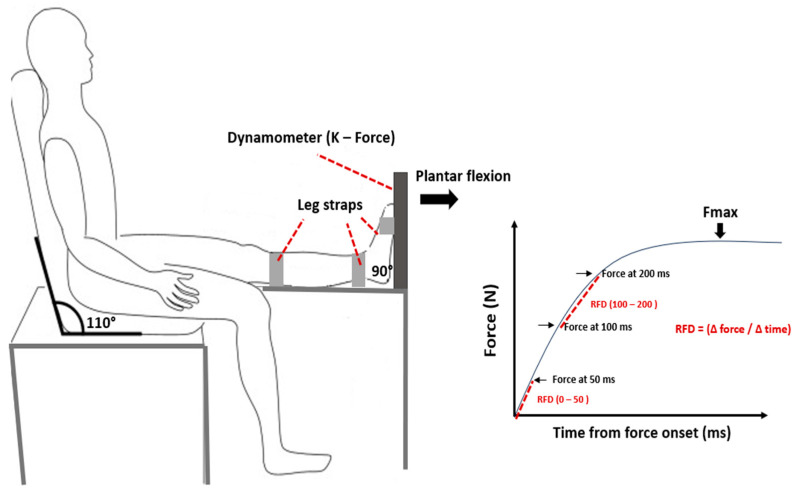
Setup for the maximal voluntary contraction test of the plantar flexors and parameter extraction.

Neuromuscular parameters of PF of the dominant leg were assessed during maximal voluntary contractions using a dynamometer (K-Force, Kinvent, Montpellier, France). Participants were seated on a chair, ensuring contact between their back, buttocks, and thighs with the chair while keeping their legs horizontally extended (Figure 2). They were instructed to push with the ends of their feet against the dynamometer [22]. Two trials were conducted with a one-minute rest interval between them, and the maximum force of the PF (Fmax, N) from both trials was recorded. The relative maximum force (Fmax relative, N/kg) was calculated by normalizing the maximum force to the participant’s body mass. The early RFD was calculated from the onset of each maximal voluntary contraction to 50 ms (RFD 0–50), and the late RFD was calculated between 100 and 200 ms (RFD 100–200). Both early and late RFD were derived from the linear slope of the force–time curve (Δ force/Δ time).

### 2.4. Resistance Training Programs

Resistance training programs consisted of three sessions per week over a 12-week period, totaling 36 sessions. While both groups performed identical PF resistance exercises, the execution parameters differed significantly in cadence and stimuli (Table 1). The exercises included seated calf raises performed on a calf raise machine and toe presses conducted on a leg press machine. The EXG completed three to five sets of 12 to 14 repetitions at 40–45% of one-repetition maximum, emphasizing a rapid concentric phase followed by a controlled 3 s eccentric phase. In contrast, the STG performed three to four sets of six to seven repetitions at 80–85% of 1-RM, with both concentric and eccentric phases executed at a steady 3 s cadence. This differentiation in cadence and load was designed to isolate explosive and maximal strength training stimuli. The protocol was adapted from established studies [23,24], beginning with a two-week conditioning phase to prepare participants for the specialized training phase, which focused on the cadence- and stimuli-specific objectives of each group. Detailed methodologies for each training protocol are outlined in Table 1.

During the conditioning phase, the two groups were introduced to the specific exercises they would be performing in the subsequent specialized training phase. This period was designed to familiarize participants with the correct execution of each movement, emphasizing proper technique and the controlled application of force. Using light loads, participants practiced the exercises with a focus on mastering the concentric and eccentric phases. The EXG was instructed to perform the concentric phase rapidly while maintaining control during the eccentric phase, whereas the STG was guided to execute both phases in a slow and controlled manner. During the final session of the conditioning phase, participants underwent a one-maximal repetition assessment using a seated calf raise machine. This assessment involved a progressive increase in load, with small incremental increases, until participants achieved the maximum weight they could lift with proper form. The one-maximal repetition was defined as the heaviest load lifted with controlled execution and without any compensatory movements, and it was found to be approximately 22 kg for both groups.

To systematically increase the workload, the specialized training phase was organized into five micro-cycles, each spanning two weeks. To ensure that the relative load training between the EXG and STG groups was equalized, the workload was quantified using the volume load method [23]. The volume load was calculated using the following equation [25]:

Volume load = number of sets × number of repetitions × weight lifted (kg)(2)

Table 2 presents the predicted progression of volume-equated training loads between the two groups. The predicted volume loads were established based on the first 1–maximal repetition assessment.

To ensure optimal progression throughout the program, one-maximal repetition assessments were conducted at the end of each micro-cycle [23], with the volume load systematically adjusted based on participants’ performance. These adjustments were made at the end of each week, following the final training session, to maintain an appropriate and challenging stimulus for continued adaptation [23,26]. A standardized warm-up and cool-down were integrated into each training session. The warm-up consisted of 5–10 min of low-intensity activity (e.g., walking or cycling). The cool-down included static stretching of the major lower limb muscle groups and 2 min of light walking to facilitate recovery.

Throughout the 10 weeks of the specialized training phase, the training load was assessed after each session using the rate of perceived exertion scale [27]. The average daily training load for each group was calculated using the following equation:

(3) Daily training load=Rate of perceived exertion×volume load,

Training monotony and strain of each group was calculated based on the formulas proposed by Foster et al. [28]:

Monotony = Weakly mean training load/SD, (4)

Strain = Total weekly training load × Monotony,(5)

where the weekly training load is the average daily training load during the week, and SD is the standard deviation of the daily training load calculated over a week. The recovery durations between repetitions and sets were carefully selected based on established guidelines and empirical evidence from previous studies [23,29,30]. By maintaining equal recovery times for both training modalities, we aimed to standardize training volume and workload, thereby allowing for a rigorous comparison of their specific effects on neuromuscular and functional outcomes.

### 2.5. Statistical Analysis

The sample size was determined using the freeware GPower (version 3.1.9.4), as outlined in the study by Ferhi et al. [21], based on a priori power analysis for an ANOVA test with repeated measures. The calculation assumed a moderate effect size (f = 0.25), a significance level of α = 0.05, and a statistical power (1 − β) of 0.80 to detect significant differences between groups and over time. The primary variable used for this calculation was PF strength, as it is a key determinant of functional outcomes such as gait speed in older adults. Based on these parameters, a minimum of 40 participants were required to ensure sufficient power. To account for an expected attrition rate of 20%, derived from previous studies on resistance training interventions in older adults [23,29], the target recruitment was set at 50 participants. The clinically significant change was defined as a 10% improvement in gait speed and PF strength, based on thresholds established in prior research as meaningful for enhancing mobility and reducing fall risk in older adults [21,31].

Statistical analysis was performed using Statistica Software 13.0 (StatSoft, Tulsa, OK, USA). The normality of the data sets and the homogeneity of variances were assessed using the Shapiro–Wilk and Levene’s tests, respectively. The effects of time (pre- and post-training) and group (EXG and STG) on neuromuscular and functional parameters, as well as their interaction, were tested using a two-factor ANOVA (group × time). Subsequently, a post hoc analysis was conducted to determine significant inter- and intra-group effects. Finally, Pearson correlation analysis (r) was performed to examine the relationship between walking speed and the neuromuscular parameters of the PF before and after interventions. Results at baseline and after the intervention were presented as mean ± standard deviation. The significance level was set at *p* < 0.05.

## 3. Results

### 3.1. Participants

A total of 57 volunteers were initially recruited for this study (Figure 2). However, only 46 participants met the established eligibility criteria and were randomly assigned to either the EXG (n = 23) or the STG (n = 23). Six participants did not complete the study due to non-adherence to the protocol—five from the EXG and one from the STG. Reasons for discontinuation included hospitalizations due to stroke, hip fracture, and ankle sprain in four participants, while two others withdrew for personal reasons. Consequently, adherence to the study protocol was 87%. Ultimately, a cohort of 40 participants successfully completed the study (Table 3), with the EXG consisting of 18 participants (age = 80.41 ± 10.12 years; body mass index = 22.89 ± 2.77 kg/m^2^) and the STG consisting of 22 participants (age = 82.89 ± 5.32 years; body mass index = 23.81 ± 3.45 kg/m^2^).

### 3.2. Training Programs

**Figure 3:**
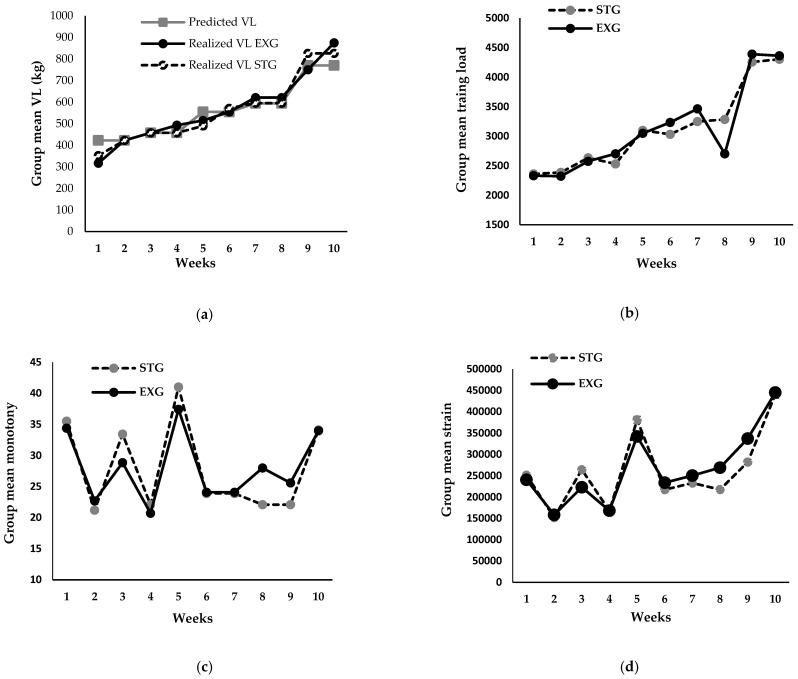
Comparative analysis of training metrics over the specialized training phase (10 weeks). (**a**) Predicted and realized volume load in explosive (EXG) and strength (STG) Groups. (**b**) Weekly progression of training load in explosive (EXG) and strength (STG) Groups. (**c**) Weekly training monotony in explosive (EXG) and strength (STG) Groups. (**d**) Weekly training strain in explosive (EXG) and strength (STG) Groups.

Attendance was calculated as the average percentage of training sessions attended over the 12-week period. Overall, attendance was 91.9 ± 3.5% for both the EXG and STG, with no statistically significant differences between the groups in session attendance. The one-maximal repetition values ranged from 22 kg to 25 kg in the EXG and from 22 kg to 27 kg in the STG. Overall, no statistically significant differences were observed between the predicted volume load and the realized volume load in both groups. A notable deviation occurred during the first week, where the predicted load was overestimated by 20% for the EXG and by 15% for the STG. Conversely, in the 10th week, the load was underestimated by 13% for EXG and 7% for STG (Figure 3a). Except for the 8th week, no significant differences in group mean training load (Figure 3b), monotony (Figure 3c), and strain (Figure 3d) were observed between the two groups. Monotony values for both groups ranged from 22 to 42 (Figure 3c), indicating that the variability in training load was effectively managed, thereby preventing excessive uniformity that could potentially lead to overtraining. The similar strain values further indicate that the overall training stimulus was appropriately balanced, promoting analogous adaptation processes in both groups.

### 3.3. Anthropometric Parameters

No significant inter- or intra-group differences were observed between the STG and EXG groups, both before and after training, across all anthropometric parameters presented in Table 3.

### 3.4. Walking Speed

**Figure 4:**
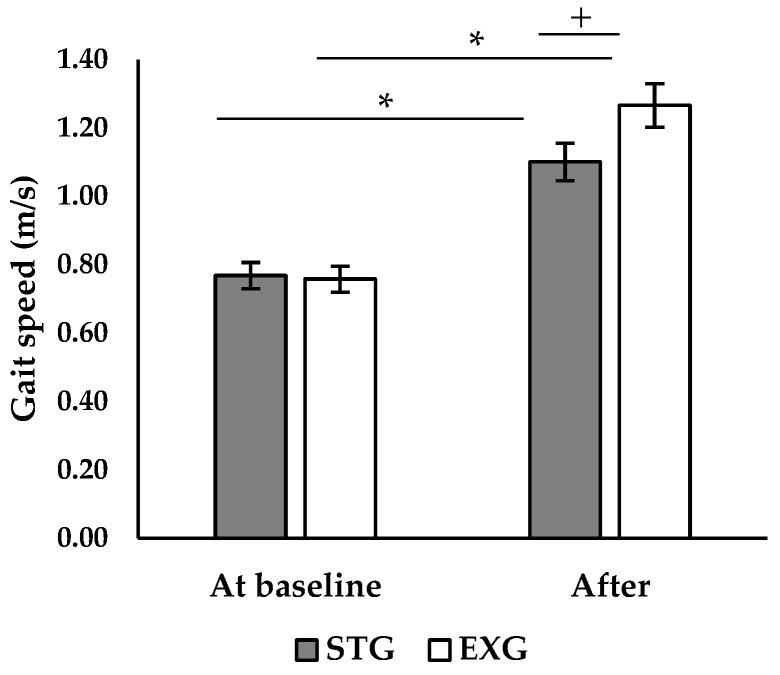
Comparison of walking speed between the two groups before and after the intervention. *: significant difference between before and after the intervention (*p* < 0.05); +: significant difference between groups (*p* < 0.05).

ANOVA analysis revealed a significant interaction between the effect of time and group on walking speed (*p* < 0.001; F = 11.6). Post hoc analysis showed that there was no significant difference between the two groups before the intervention (Figure 4). After the intervention, both EXG and STG showed a significant increase in walking speed (+63.2%, *p* < 0.001; +45.6%, *p* < 0.001, respectively). However, walking speed was higher in the EXG compared to the STG (*p* < 0.05, Figure 4).

### 3.5. Neuromuscular Parameters

ANOVA analysis revealed a significant interaction between the effects of time and group (Table 4) on both absolute (*p* < 0.001; F = 1.77) and relative Fmax (*p* < 0.001; F = 14.6). Post hoc analysis revealed a significant increase in both absolute and relative Fmax in the EXG (+20.5%, +21.1%, *p* < 0.001, respectively) and in the STG (+16.2%, +17.1%; *p* < 0.001, respectively). The improvement in both absolute and relative Fmax was greater in EXG (*p* < 0.05). Additionally, post hoc analysis showed a significant increase in RFD 0–50 of 10% in the STG (*p* < 0.05) and 20.1% (*p* < 0.05) in the EXG. The improvement in RFD 0–50 was greater in the EXG (*p* < 0.05). Moreover, RFD 100–200 improved only in the EXG (+20.4%, *p* < 0.05).

### 3.6. Relationship Between Neuromuscular Parameters of the PF and Walking Speed

#### 3.6.1. At Baseline

**Figure 5:**
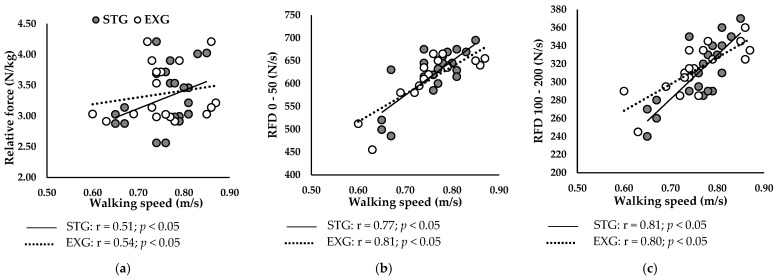
Relationships between walking speed and neuromuscular parameters of plantar flexors at baseline. (**a**) Relationship between gait speed and relative maximal force. (**b**) Relationship between gait speed and RFD 0–50. (**c**) Relationship between gait speed and RFD 100–200.

Figure 5 presents correlations between walking speed and neuromuscular parameters at baseline in STG and EXG. Walking speed was positively correlated with the relative Fmax (Figure 5a) of STG (r = 0.51; *p* < 0.05) and EXG (r = 0.54; *p* < 0.05).

Additionally, RFD 0–50 (Figure 5b) and RFD 100–200 (Figure 5c) were positively correlated with walking speed in STG (r = 0.77; r = 0.81; *p* < 0.05, respectively) and in EXG (r = 0.81; r = 0.80; *p* < 0.05, respectively).

#### 3.6.2. After Training Programs

**Figure 6:**
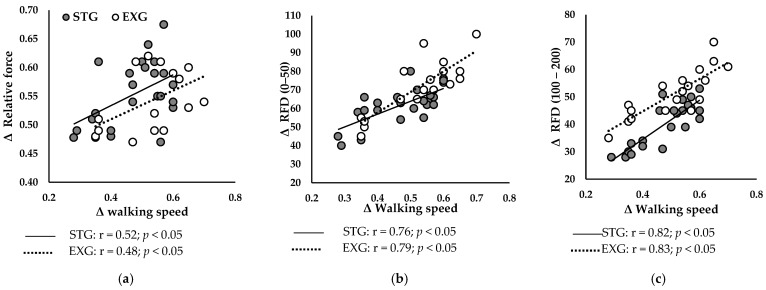
Relationships between ameliorations in gait speed and improvements in neuromuscular parameters of plantar flexors. (**a**) Relationship between Δ gait speed and Δ relative maximal force. (**b**) Relationship between Δ gait speed and Δ RFD 0–50. (**c**) Relationship between Δ gait speed and Δ RFD 100–200.

Figure 6 presents correlations between ameliorations in walking speed and improvements in neuromuscular parameters in STG and EXG. Δ walking speed was positively correlated with Δ relative Fmax (Figure 6a) of STG (r = 0.52; *p* < 0.05) and EXG (r = 0.48; *p* < 0.05). Additionally, Δ RFD 0–50 (Figure 6b) and Δ RFD 100–200 (Figure 6c) were positively correlated with Δ walking speed in STG (r = 0.76; r = 0.82; *p* < 0.05, respectively) and in EXG (r = 0.79; r = 0.83; *p* < 0.05, respectively).

## 4. Discussion

This study aimed to evaluate the effects of explosive versus strength resistance training on the neuromuscular parameters of the PF, as well as to examine the relationships between improvements in these parameters and walking speed in institutionalized older adults. Our results revealed that both types of resistance training improved the neuromuscular capacities of the PF but with different and specific adaptations. Strength resistance training led to a more significant improvement in Fmax, while explosive training favored improvements in RFD. Furthermore, regardless of the type of training, improvement in walking speed was more strongly correlated with RFD in both intervention groups.

The results of this study revealed that resistance training programs, regardless of the stimuli and cadence (explosive or strength), improved the Fmax of PF. These findings are consistent with several studies, highlighting possible neuromuscular adaptations related to resistance exercises, allowing for significant improvements after 12 weeks of muscle resistance training [14,32]. Specifically, our results showed a 21% improvement in relative Fmax in STG and 16% in the EXG. These results align with the systematic review of Lopez et al. [33], showing significant improvements in maximum knee extensor force ranging from 6.6% to 37.0%, after 12 weeks of resistance training in older adults.

Several hypotheses can be proposed to explain the mechanisms of these neuromuscular adaptations to resistance training in older adults. One key aspect involves biochemical markers, specifically those belonging to the transforming growth factor β (TGF-β) superfamily [34]. This superfamily plays a central role in regulating muscle architecture by orchestrating extracellular matrix remodeling and cellular proliferation processes [35]. Within this group, growth differentiation factor-8 (GDF-8, also known as myostatin), growth differentiation factor-15 (GDF-15), and activin A are recognized as potent negative regulators of muscle homeostasis, often associated with muscle atrophy in humans [36]. On the other hand, follistatin acts as a critical antagonist to myostatin, promoting muscle hypertrophy and maintaining muscle mass [35]. In this context, Seo et al. [36] examined the effects of a 16-week resistance training program on muscle growth factors using blood sample analyses. Their findings revealed that only follistatin levels showed a significant increase (3.1%) in the resistance training group, whereas no significant changes were observed in the levels of GDF-8, GDF-15, or activin A. This selective increase in follistatin underscores its pivotal role in counteracting myostatin’s inhibitory effects on muscle growth, thereby facilitating resistance training-induced improvements in muscle strength and function. These findings suggest that the observed neuromuscular adaptations to resistance training may primarily stem from the upregulation of myostatin-inhibitory mechanisms mediated by follistatin. Additionally, the absence of changes in GDF-8, GDF-15, or activin A may reflect the specificity of the training protocol used in the study. It is possible that these markers are less sensitive to moderate resistance training or require more prolonged and intense exercise interventions to elicit significant responses.

In addition to molecular changes, structural adaptations may also contribute to the observed increases in muscle strength. Specifically, increases in the cross-sectional area (CSA) of muscle fibers through hypertrophy are often associated with enhanced force production (Fmax). For instance, Kryger et al. [32] demonstrated a significant 22% increase in the number of type IIa muscle fibers in the knee extensor muscles of older adults, promoting muscle hypertrophy linked to improved Fmax. However, in this study, no significant increase in lean mass was observed in either group following the intervention. This finding is consistent with Bardstu et al. [37], reporting no significant changes in body composition after eight months of resistance training in older adults. These results suggest that strength gains may primarily result from improvements in muscle quality rather than muscle mass. Potential mechanisms include a reduction in fat infiltration within the muscle [14] and neural adaptations, such as increased motor unit recruitment and enhanced nerve transmission efficiency [38]. These findings underscore the role of neuromuscular adaptations in improving muscle function and highlight the effectiveness of resistance training in mitigating age-related declines in muscle quality.

The originality of this study lies in its investigation of specific adaptations associated with cadence and stimuli of resistance exercises in older adults. Our results show that strength resistance training led to a greater improvement in Fmax (+8% in STG), while explosive resistance training produced more pronounced improvements in early RFD (+9% in EXG) and late RFD (+8% in EXG). These findings align with previous studies highlighting the distinct neuromuscular demands of explosive and strength training protocols in older adults [39,40]. Explosive training appears to target neural mechanisms influencing early RFD, such as motor unit recruitment and discharge rates [9,10]. Early-phase improvements are likely driven by enhanced neural activation, including reduced cortical inhibition and improved nerve conduction velocity [14]. Improvements in late RFD likely reflect structural adaptations within the muscle-tendon complex, including increased tendon stiffness and enhanced force transmission from the contractile elements through the parallel (e.g., extracellular matrix) and series elastic components (e.g., tendons) to the joint articulation [38]. Additionally, potential structural changes, such as increased pennation angle and muscle thickness, may further optimize the muscle’s mechanical efficiency during force production [41,42]. These adaptations collectively contribute to improved force output and resilience in dynamic movements.

Our results revealed a significant improvement in walking speed for both EXG (+63.2%) and STG (+45.6%), consistent with several studies showing similar effects of muscle resistance training on habitual and maximum walking speed [33,43]. These improvements, ranging from 5.5% to 20.4% and observed after short-term interventions (10-12 weeks), have been attributed to enhanced neuromuscular capacities of lower limb muscles [14]. However, in our study, the EXG showed a significantly superior improvement of 12% in walking speed compared to the STG. Moreover, the improvement in walking speed was more strongly correlated with the RFD than with the improvement in Fmax (Figure 6). Indeed, during walking, the time to develop force is limited, typically less than 300 ms. Superior gains in RFD, both in the early (0–50 ms) and late (100–200 ms) phases, allow for a rapid and efficient response, essential for maintaining balance and stability during walking [38,44]. Additionally, improved RFD enhances propulsion and braking during walking, crucial for speed and movement efficiency [45]. These results suggest that improvements in RFD play a more decisive role in enhancing walking propulsion capacity than increases in Fmax. This underscores the importance of targeting these parameters in rehabilitation and training programs to optimize activities of daily living in older adults [37,46]. Moreover, an increased ability to rapidly develop force allows for better responses to situations of imbalance, such as during stumbling, and helps maintain equilibrium and stability while walking [11,47]. Therefore, rehabilitation programs should include explosive resistance exercises to maximize neuromuscular and functional benefits in older adults.

### Limitations and Future Perspectives

This study acknowledges several limitations that warrant consideration. Firstly, our study primarily focused on the PF. It would be important to investigate other muscle groups, especially those around the knee, which are also crucial for walking. An analysis of neuromuscular activities through electromyography would also be necessary to understand the underlying mechanisms of neural adaptations resulting from the two types of interventions. Finally, while bioelectrical impedance analysis is a practical and widely used method for estimating body composition due to its non-invasive nature, portability, and ease of use, it relies on population-specific algorithms that may not be universally applicable to our study population. In future studies, it would be beneficial to incorporate gold-standard techniques, such as Magnetic Resonance Imaging or Dual-Energy X-ray Absorptiometry for assessing body composition.

## 5. Conclusions

The cadence and stimuli of muscle resistance exercises elicit distinct neuromuscular adaptations in older adults. Explosive resistance training appears particularly effective in improving the ability to rapidly generate force, which is essential for many daily activities requiring explosive movements and quick responses. These results underscore the importance of including explosive resistance exercises, supported by regular assessments and adjustments based on individual progress, to maximize neuromuscular and functional benefits in older adults.

## Figures and Tables

**Table 1:** Details and methodologies employed in each training protocol.

	Explosive Resistance Training	Strength Resistance Training
Sets	3 to 5 sets	3 to 4 sets
Repetitions	12 to 14 repetitions	6 to 7 repetitions
Intensity	40% to 45% of 1–1-maximal repetition	80% to 85% of 1–1-maximal repetition
Concentric Phase Execution	rapidly	≈3 s
Eccentric Phase Execution	≈3 s	≈3 s
Recovery Between Repetitions	1 s	1 s
Recovery Between Sets	5 min between sets	5 min between sets

**Table 2:** Predicted progression of volume load of resistance training programs.

Week	Micro-Cycle	Explosive Resistance Training	Strength Resistance Training	Volume Load (kg)
1–2	1	conditioning phase	conditioning phase	-
3–4	2	4 sets × 12 reps @ 40% 1-RM (8.8 kg)	3 sets × 8 reps @ 80% 1-RM (17.6 kg)	422.4 kg each
5–6	3	4 sets × 13 reps @ 40% 1-RM (8.8 kg)	3 sets × 9 reps @ 80% 1-RM (17.6 kg)	457.6 kg each
7–8	4	4 sets × 14 reps @ 45% 1-RM (9.9 kg)	4 sets × 7 reps @ 85% 1-RM (18.7 kg)	554.4 kg each
9–10	5	5 sets × 12 reps @ 45% 1-RM (9.9 kg)	4 sets × 8 reps @ 85% 1-RM (18.7 kg)	594.0 kg each
11–12	6	5 sets × 14 reps @ 50% 1-RM (11.0 kg)	4 sets × 9 reps @ 85% 1-RM (18.7 kg)	770.0 kg each

**Table 3:** Anthropometric characteristics of the two groups before and after the intervention.

		Mean ± SD	Mean ± SD	*p* (F)		
Parameters	Groups	At Baseline	After	Time	Time × Group	Group
Age (years)	STG	82.89 ± 5.32	82.89 ± 5.32	0.92 (0.48)	0.91 (0.01)	0.76 (0.09)
	EXG	80.41 ± 10.12	80.41 ± 10.12			
Height (m)	STG	1.70 ± 0.13	1.70 ± 0.13	0.84 (0.69)	0.792 (0.07)	0.92 (0.16)
	EXG	1.64 ± 0.16	1.64 ± 0.16			
Weight (kg)	STG	68.81 ± 5.60	70.43 ± 7.60	0.76 (0.85)	0.746 (0.03)	0.81 (0.02)
	EXG	61.62 ± 9.45	64.19 ± 4.45			
Body mass index (kg/m^2^)	STG	23.81 ± 3.45	24.39 ± 3.34	0.71 (0.85)	0.746 (0.32)	0.87 (0.24)
	EXG	22.89 ± 2.77	23.78 ± 3.23			
Lean mass (kg)	STG	44.90 ± 4.74	48.34 ± 2.71	0.54 (0.48)	0.908 (0.01)	0.76 (0.09)
	EXG	41.65 ± 3.65	44.94 ± 5.64			
Fat mass (kg)	STG	23.91 ± 9.26	22.09 ± 7.28	0.95 (0.69)	0.792 (0.07)	0.90 (0.02)
	EXG	19.97 ± 4.60	19.25 ± 6.55			
Fat mass (%)	STG	34.76 ± 3.04	31.38 ± 5.10	0.79 (0.75)	0.75 (0.03)	0.87 (0.02)
	EXG	32.40 ± 6.15	29.99 ± 7.16			

STG: strength resistance training group; EXG: explosive resistance training group; SD: standard deviation.

**Table 4:** Neuromuscular parameters of the two groups before and after interventions.

		Mean ± SD	Mean ± SD	*p* (F)		
Parameters	Groups	At Baseline	After	Time	Time × Group	Group
Absolute Fmax (N)	STG	220.00 ± 13.69	265.00 ± 13.69 *	<0.001 (113.00)	<0.001 (81.77)	0.130 (2.53)
	EXG	216.50 ± 9.44	251.50 ± 9.44 *+			
Relative Fmax (N/kg)	STG	3.45 ± 0.53	4.177 ± 0.61 *	<0.001 (973.71)	0.001 (14.62)	0.376 (0.83)
	EXG	3.31 ± 0.45	3.875 ± 0.53 *+			
RFD 0–50 (N/s)	STG	630.00 ± 41.07	693.22 ± 45.23 *	<0.001 (625.51)	0.07 (8.71)	0.106 (2.92)
	EXG	627.50 ± 27.00	753.40 ± 32.87 *+			
RFD 100–200 (N/s)	STG	320.00 ± 27.38	352.00 ± 30.12	<0.001 (908.95)	<0.001 (107.70)	0.202 (1.76)
	EXG	318.00 ± 18.88	383.60 ± 19.99 *+			

STG: strength resistance training group; EXG: explosive resistance training group; SD: standard deviation. *: significant difference between before and after the intervention (*p* < 0.05); +: significant difference between the two groups (*p* < 0.05).

## Data Availability

The research was registered in the Pan African Clinical Trials Registry under the registration number PACTR202306912191110.
